# The Role of Artificial Intelligence and Machine Learning in Cardiovascular Imaging and Diagnosis

**DOI:** 10.7759/cureus.68472

**Published:** 2024-09-02

**Authors:** Setareh Reza-Soltani, Laraib Fakhare Alam, Omofolarin Debellotte, Tejbir S Monga, Vaishali Raj Coyalkar, Victoria Clarice A Tarnate, Chioma Ugochinyere Ozoalor, Sanjana Reddy Allam, Maham Afzal, Gunjan Kumari Shah, Manju Rai

**Affiliations:** 1 Advanced Diagnostic & Interventional Radiology Center (ADIR), Tehran University of Medical Sciences, Tehran, IRN; 2 Internal Medicine, Ministry of Health, Kuwait City, KWT; 3 Internal Medicine, One Brooklyn Health-Brookdale Hospital Medical Center, Brooklyn, USA; 4 Internal Medicine, Spartan Health Sciences University, Vieux Fort, LCA; 5 Radiodiagnosis, Malla Reddy Institute of Medical Sciences, Hyderabad, IND; 6 Medicine, Far Eastern University - Dr. Nicanor Reyes Medical Foundation, Quezon City, PHL; 7 Internal Medicine, Worcestershire Royal Hospital, Worcester, GBR; 8 Internal Medicine, Gandhi Medical College, Secunderabad, IND; 9 Medicine, Fatima Jinnah Medical University, Lahore, PAK; 10 Internal Medicine, Janaki Medical College, Janakpurdham, NPL; 11 Biotechnology, Shri Venkateshwara University, Gajraula, IND

**Keywords:** personalized medicine, cardiomyopathy, coronary artery disease, diagnostic accuracy, magnetic resonance imaging, computed tomography, echocardiography, cardiovascular imaging, machine learning, artificial intelligence

## Abstract

Cardiovascular diseases remain the leading cause of global mortality, underscoring the critical need for accurate and timely diagnosis. This narrative review examines the current applications and future potential of artificial intelligence (AI) and machine learning (ML) in cardiovascular imaging. We discuss the integration of these technologies across various imaging modalities, including echocardiography, computed tomography, magnetic resonance imaging, and nuclear imaging techniques. The review explores AI-assisted diagnosis in key areas such as coronary artery disease detection, valve disorders assessment, cardiomyopathy classification, arrhythmia detection, and prediction of cardiovascular events. AI demonstrates promise in improving diagnostic accuracy, efficiency, and personalized care. However, significant challenges persist, including data quality standardization, model interpretability, regulatory considerations, and clinical workflow integration. We also address the limitations of current AI applications and the ethical implications of their implementation in clinical practice. Future directions point towards advanced AI architectures, multimodal imaging integration, and applications in precision medicine and population health management. The review emphasizes the need for ongoing collaboration between clinicians, data scientists, and policymakers to realize the full potential of AI in cardiovascular imaging while ensuring ethical and equitable implementation. As the field continues to evolve, addressing these challenges will be crucial for the successful integration of AI technologies into cardiovascular care, potentially revolutionizing diagnostic capabilities and improving patient outcomes.

## Introduction and background

Cardiovascular diseases (CVDs) remain the leading cause of mortality worldwide, accounting for an estimated 31% of all deaths globally [1]. The profound impact of CVDs on public health and healthcare systems underscores the critical need for accurate and timely diagnosis. Cardiovascular imaging plays a pivotal role in this diagnostic process, providing essential insights into cardiac structure and function [2].

Traditional cardiovascular imaging techniques such as echocardiography, computed tomography (CT), magnetic resonance imaging (MRI), and nuclear imaging have been instrumental in identifying and characterizing various cardiac conditions [3]. However, these methods often face challenges, including inter-observer variability, time-consuming image analysis, and the potential for missed subtle abnormalities [4]. Moreover, the increasing volume and complexity of imaging data have created a need for more efficient and accurate interpretation methods.

**Figure 1:**
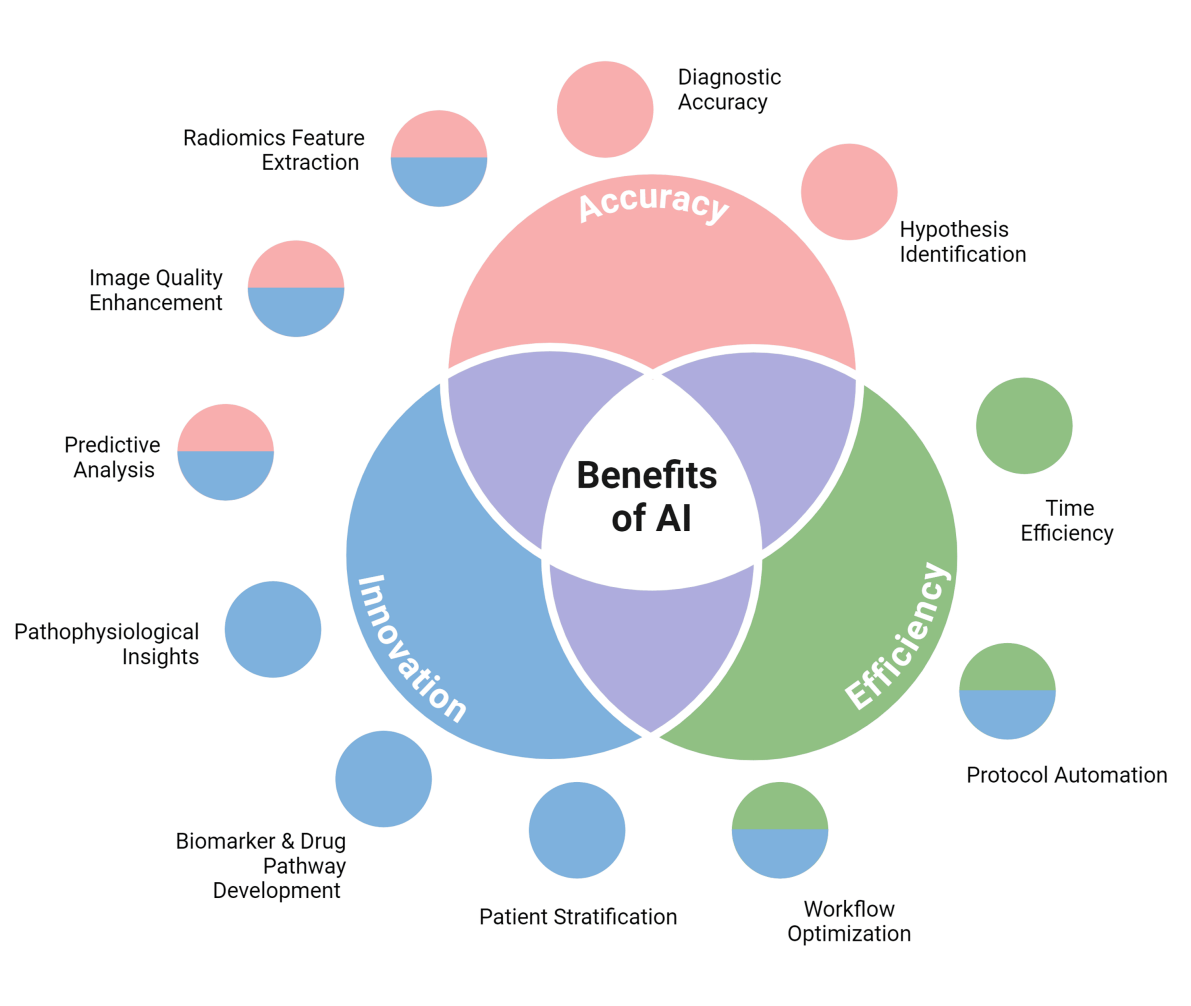
Advantages of integration of artificial intelligence (AI) and machine learning into medical imaging Image Credits: Omofolarin Debellotte; image created in BioRender.com

In recent years, the integration of artificial intelligence (AI) and machine learning (ML) into medical imaging has emerged as a transformative force, promising to revolutionize the field of cardiology [5]. These innovative technologies offer the potential to enhance image acquisition, streamline data analysis, improve diagnostic accuracy, and ultimately lead to more personalized patient care (Figure 1). As the volume and complexity of cardiovascular imaging data continue to grow exponentially, AI and ML algorithms present advanced solutions to interpret and leverage this wealth of information, potentially uncovering novel insights and patterns beyond human perception [6].

The application of AI and ML in cardiovascular imaging addresses several key challenges in current diagnostic methods. These technologies can reduce inter-observer variability, accelerate image analysis, and potentially identify subtle features that might be overlooked by human observers [7]. Furthermore, AI and ML can integrate and analyze large datasets from multiple imaging modalities, providing a more comprehensive view of a patient's cardiovascular health [8].

The objectives of this narrative review are twofold. First, we aim to provide a comprehensive overview of the current applications and potential future developments of AI and ML in cardiovascular imaging and diagnosis. Second, we seek to critically evaluate the challenges, limitations, and ethical considerations associated with the implementation of these technologies in clinical practice.

## Review

AI and ML technologies in cardiovascular imaging

AI encompasses a broad range of computational techniques that enable machines to perform tasks typically requiring human intelligence [9]. ML, a subset of AI, involves algorithms that can learn from and make predictions or decisions based on data [10]. In the context of cardiovascular imaging, these technologies are being applied to various modalities, including echocardiography, CT, MRI, and nuclear imaging [4].

Deep learning, a subset of ML, has gained significant traction in medical imaging due to its ability to automatically learn hierarchical representations of data [11]. Convolutional neural networks (CNNs) are a type of deep learning architecture particularly well-suited for image analysis [12]. CNNs consist of multiple layers that can automatically extract relevant features from images, making them highly effective for tasks such as image classification, segmentation, and object detection in cardiovascular imaging [13].

In echocardiography, CNNs have been successfully employed for automated view classification, left ventricular segmentation, and measurement of cardiac function parameters [14]. For cardiac CT, deep learning algorithms have shown promise in coronary artery calcium scoring, plaque characterization, and coronary stenosis detection [15]. In cardiac MRI, CNNs have been applied to tasks such as automated segmentation of cardiac chambers and structures, tissue characterization, and perfusion analysis [16].

While deep learning has garnered significant attention, other ML algorithms also play important roles in cardiovascular imaging. Support vector machines (SVMs) have been used for image classification and risk stratification tasks [17]. Random forests have shown utility in feature selection and prediction of cardiovascular events based on imaging biomarkers [18]. Clustering algorithms, such as k-means, have been applied to identify patterns in imaging data that may correspond to different disease phenotypes [19].

Unsupervised learning techniques, including autoencoders and generative adversarial networks (GANs), have demonstrated potential in image denoising, super-resolution, and synthetic data generation for cardiovascular imaging [20]. These approaches can help improve image quality, reduce radiation dose, and address data scarcity issues in ML model development.

As AI and ML technologies continue to advance, their integration into clinical cardiovascular imaging workflows presents both opportunities and challenges. While these tools show great promise in improving diagnostic accuracy and efficiency, careful validation, regulatory considerations, and ethical implications must be addressed to ensure their safe and effective implementation in patient care [21].

Applications of AI in cardiovascular imaging modalities

**Figure 2:**
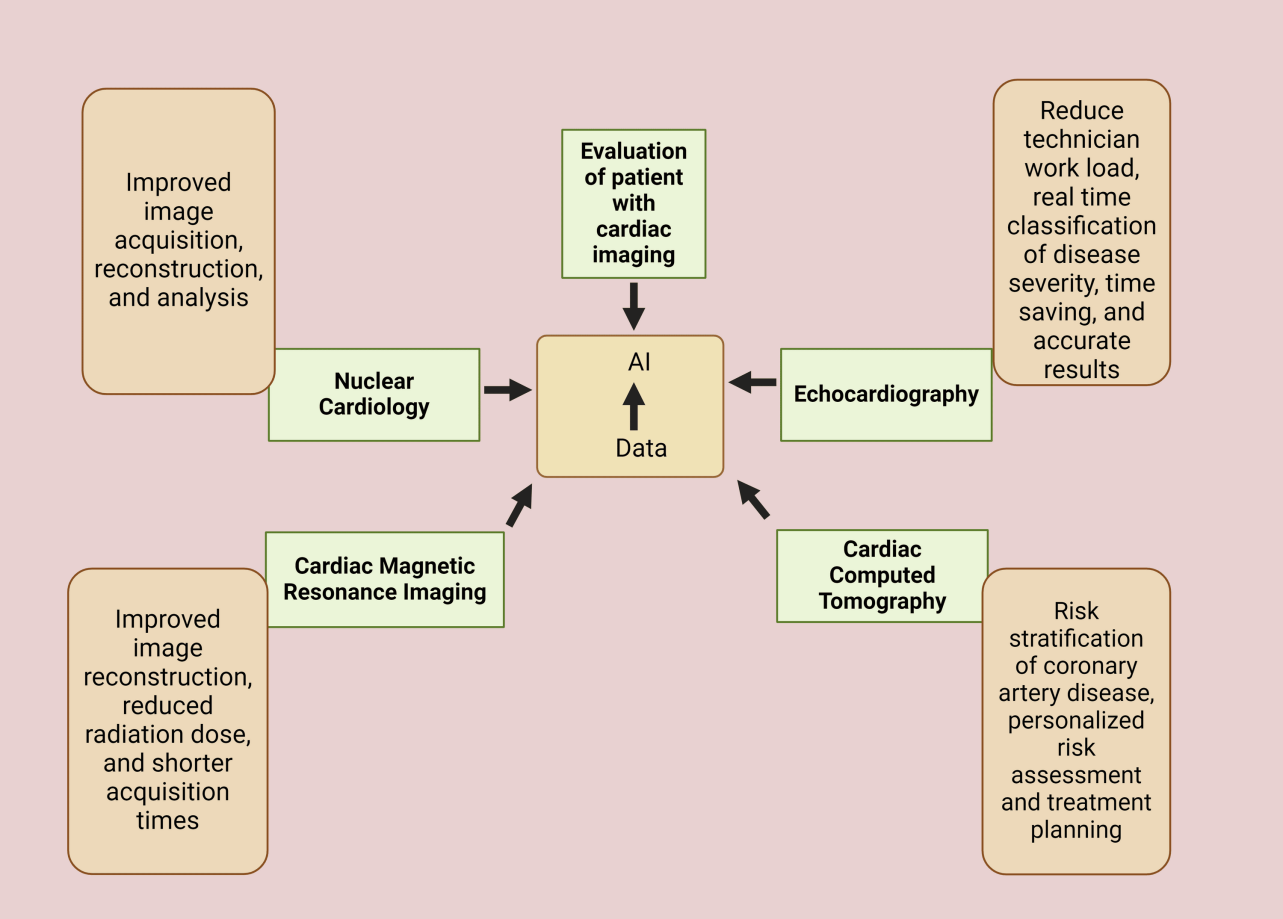
Cardiac imaging modalities collect data that serves as the basis for developing artificial intelligence (AI) used to optimize the evaluation of patients undergoing cardiac imaging Image Credit: Maham Afzal; image created in BioRender.com

**Figure 3:**
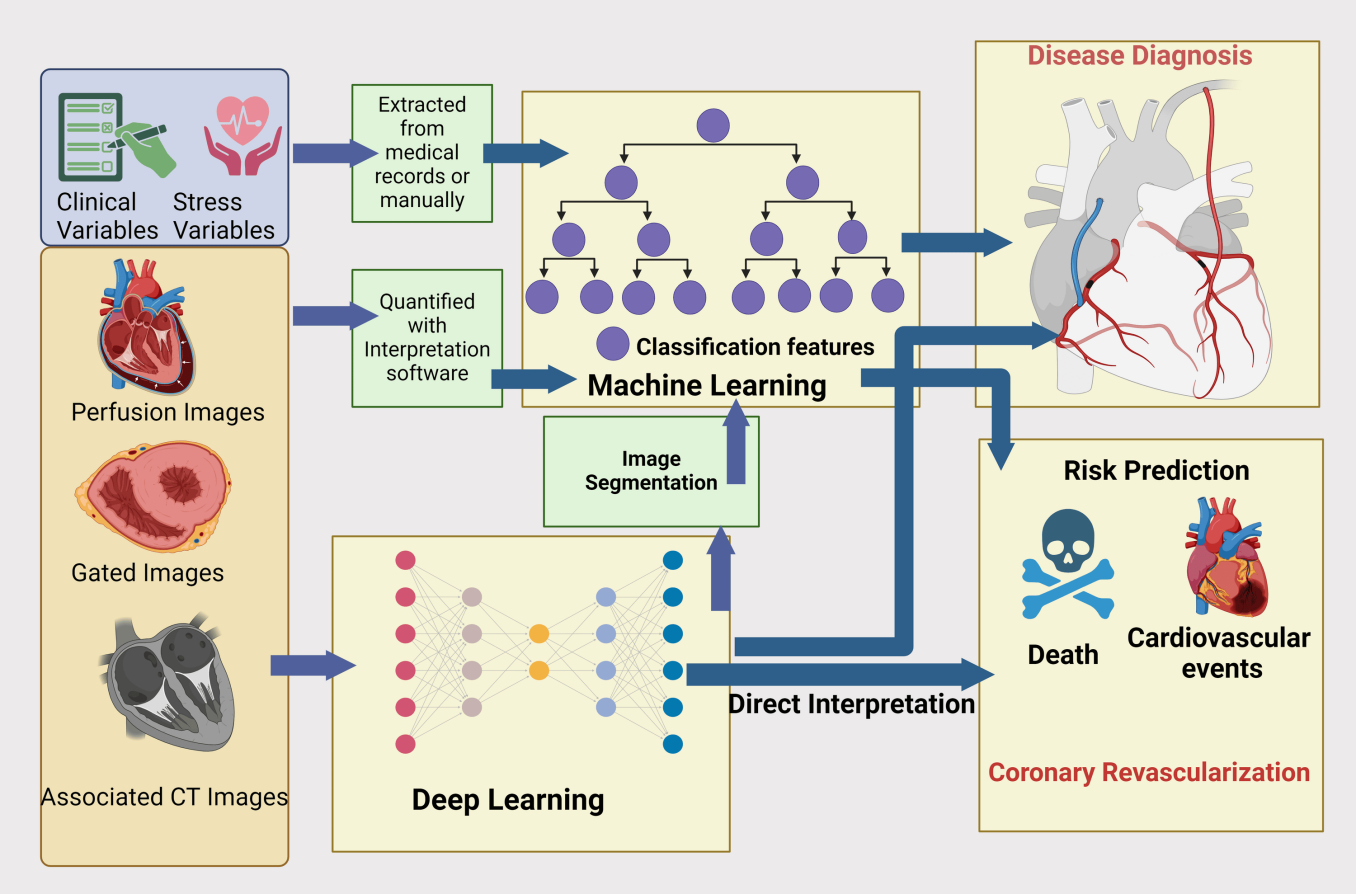
Role of machine learning and deep learning in risk prediction and disease diagnosis in cardiovascular events Image Credit: Gunjan Kumari Shah; image created in BioRender.com

AI has shown remarkable potential across various cardiovascular imaging modalities, enhancing diagnostic accuracy, efficiency, and patient care (Figure 2 and Figure 3).

Echocardiography

The rising prevalence of heart failure in aging populations is driving the demand for echocardiography, the primary method for evaluating cardiac function. Echocardiography requires trained sonographers and cardiologists to obtain and interpret images [22]. However, a shortage of highly trained professionals leads to delays in diagnosis and treatment, resulting in poorer patient outcomes [23]. AI is expected to play a significant role in addressing the inconsistency and variability in image acquisition and interpretation among healthcare workers [24].

Echocardiography helps assess chamber size, wall motion, valvular function, and, crucially, left ventricular ejection fraction (LVEF). AI-based ML has demonstrated similar accuracy to expert visual determination in assessing ejection fraction [25]. The integration of AI into echocardiography has shown promising results, reducing the time to acquire and process images for LV volumes and EF determination by 77% compared to standard care [26]. This technology can significantly reduce technicians' workload while providing real-time classification of disease severity [25]. As AI continues to evolve, it has the potential to improve the efficiency and accuracy of echocardiographic assessments, ultimately enhancing patient care in the face of growing demand.

CNNs have demonstrated high accuracy in left ventricular segmentation and quantification of cardiac function parameters such as ejection fraction and strain analysis [14]. These automated measurements can save time and provide more consistent results compared to manual analysis.

AI-powered systems have also shown promise in diagnosing various cardiac conditions. For instance, ML algorithms have been developed to detect and classify valvular heart diseases with accuracy comparable to experienced cardiologists [27]. Additionally, AI models have been trained to identify patterns associated with cardiomyopathies, aiding in early diagnosis and risk stratification [28].

CT

In cardiac CT, AI has revolutionized image analysis and risk assessment. Deep learning algorithms have been developed for automated coronary artery calcium scoring, providing rapid and accurate quantification of calcification burden [29]. This application helps in risk stratification for coronary artery disease (CAD) and can guide preventive interventions. AI-based approaches have also shown excellent performance in coronary CT angiography (CCTA) for detecting and quantifying coronary artery stenosis [30]. These algorithms can automatically segment coronary arteries, identify plaques, and assess their composition, potentially reducing the time required for image interpretation and improving diagnostic accuracy.

Furthermore, AI models have been developed to extract radiomics features from cardiac CT images, enabling more precise characterization of myocardial tissue and prediction of adverse cardiac events [31]. This application of AI holds promise for personalized risk assessment and treatment planning.

MRI

AI applications in cardiac MRI have focused on improving image acquisition, reconstruction, and analysis. Deep learning techniques have been employed for automated segmentation of cardiac chambers and structures, enabling rapid and accurate quantification of cardiac function and morphology [16].

AI algorithms have also been developed for myocardial tissue characterization, including the detection and quantification of myocardial fibrosis and edema [32]. These applications can aid in the diagnosis and monitoring of various cardiomyopathies and inflammatory heart conditions. In cardiac magnetic resonance perfusion imaging, AI-based approaches have shown potential for automated quantification of myocardial blood flow and detection of ischemia [33]. These techniques can improve the accuracy and efficiency of stress perfusion analysis, potentially enhancing the diagnosis of CAD.

Nuclear Imaging Techniques

 AI has made significant contributions to nuclear cardiac imaging, particularly in image reconstruction, analysis, and interpretation. In positron emission tomography (PET), deep learning algorithms have been developed for improved image reconstruction, enabling reduced radiation dose and shorter acquisition times without compromising image quality [34].

For single-photon emission computed tomography (SPECT), AI-based approaches have shown promise in automated quantification of myocardial perfusion and detection of CAD [35]. These algorithms can provide more consistent and objective interpretations, potentially reducing inter-observer variability.

ML techniques have also been applied to integrate clinical data with nuclear imaging findings for improved risk stratification and prognosis prediction in patients with suspected or known CAD [17].

Coronary Angiography

AI applications in coronary angiography have focused on automated analysis of invasive coronary angiograms and integration with other imaging modalities. Deep learning algorithms have been developed for automated segmentation and stenosis quantification in coronary angiograms, potentially improving the accuracy and consistency of lesion assessment [36].

AI-powered systems have also shown promise in predicting fractional flow reserve (FFR) from angiographic images, potentially reducing the need for invasive FFR measurements [37]. This application could help guide revascularization decisions more efficiently and cost-effectively.

Furthermore, AI techniques have been employed to fuse information from coronary angiography with other imaging modalities, such as intravascular ultrasound (IVUS) or optical coherence tomography (OCT), for more comprehensive plaque characterization and risk assessment [24].

AI-assisted diagnosis in CVDs

*CAD Detection* 

CAD is a significant global cause of mortality and morbidity [38]. Furthermore, methods like angiography have potential issues such as allergic reactions, renal damage, and bleeding for patients, consequently, Echocardiography is commonly used as the primary diagnostic imaging method [39-40]. Several studies have been conducted on AI-assisted diagnosis of CVDs. Upton et al. developed a pipeline for automated image processing to extract new geometric and kinematic features from stress echocardiograms [38]. The results showed that the classifier achieved acceptable accuracy in identifying patients with severe CAD in the training dataset, with a specificity of 92.7% and a sensitivity of 84.4%. Furthermore, the use of the AI classification tool by clinicians led to improved inter-reader agreement, increased confidence, and heightened sensitivity in disease detection [1].

Guo et al. proposed a new method for screening CAD by utilizing ML-enhanced echocardiography, focusing on myocardial work and left atrial strain as key indicators [41]. The research involved extracting unique echocardiography features using a ML algorithm from data collected from patients undergoing coronary angiography. The study optimized a superior CAD diagnosis model using 59 echocardiographic features in a gradient-boosting classifier. The model showed a receiver operating characteristic area under the curve (AUC) value of 0.852 in the test group and 0.834 in the validation group, demonstrating high sensitivity (0.952) and low specificity (0.691), indicating its effectiveness in detecting CAD but also a potential for increased false-positive results. Additionally, the study found that false-positive cases were more likely to experience cardiac events than true-negative cases. Consequently, ML-enhanced echocardiography has the potential to improve CAD detection. 

On the other hand, detecting which coronary arteries are causing reduced blood flow in patients using only myocardial perfusion SPECT can be quite challenging [5]. Yoneyama et al. employed an artificial neural network (ANN) to analyze hybrid images that combine data from CCTA and myocardial perfusion SPECT [42]. The study showed hybrid images that integrate CCTA and myocardial perfusion SPECT data are valuable for pinpointing culprit coronary arteries. 

Valve Disorders Assessment 

Because of the specialized skills and knowledge required in the diagnosis and treatment of valvar heart disease (VHD), AI has the potential to make a big impact in this field [43]. Imaging techniques such as echocardiography, MRI, and multi-slice CT (MSCT) in confirming diagnoses related to VHD, evaluating causes, severity levels, and ventricular responses, and predicting outcomes are crucial [44]. AI is seen as beneficial for tasks like image acquisition view recognition and segmentation of structures for automated analysis. For instance, advanced algorithms can detect mitral valve conditions directly from images combining data with clinical information to uncover new subgroups and predictors related to aortic valve disease progression [43]. In addition, advanced special computer programs and software that automatically measure and map out the aortic valve help a lot when planning surgeries [7]. Moreover, AI has been playing a crucial role in VHD by using echocardiograms to suppose different types of patients and show who might be at higher risk [44].

In analyzing echocardiograms of patients with VHD, AI can ensure that the images are captured well, find the best angles, and accurately outline the valves and other heart structures for detailed analysis. Consequently, it usually focuses on four main things: getting the best images, identifying specific angles, accurately delineating structures, and identifying different disease stages [44]. 

Cardiomyopathy Classification 

Cardiomyopathies are a major cause of heart failure and dangerous heart rhythms. Finding out what causes them is crucial for treating and diagnosing these diseases. Clinicians use a mix of information like personal and family history, physical exams, electrocardiograms, lab tests, and advanced imaging that makes it hard to diagnose. Whereas, AI has shown it can find connections in lots of data and handle complex jobs better than usual methods [45]. Zhou et al. checked how well a using advanced ML program could tell the difference between two main types of cardiomyopathy: ischemic cardiomyopathy (ICM) and dilated cardiomyopathy (DCM) by echocardiogram data [46]. Furthermore, Gopalakrishnan et al. used a new approach, cardiac MRI-biomarker extraction and discovery (cMRI-BED). It uses computer tools to process images, identify markers, and make predictions. The study showed that the cMRI-BED method performed well, with a Bayesian Rule Learning (BRL) decision tree model [47]. The researchers also discovered that the presence of myocardial delayed enhancement (MDE) is an important factor in predicting cardiomyopathies, and it was effectively identified by their models. These findings suggest that the cMRI-BED framework can effectively process complex imaging data and provide valuable insights that can improve our understanding of pediatric cardiomyopathy.

Arrhythmia Detection and Classification 

ECG is the primary method for diagnosing heart rhythm issues and other cardiac conditions. Insertable cardiac monitors (ICMs) have been developed to continuously monitor heart activity over extended periods and detect four specific cardiac patterns including ventricular tachycardia, atrial tachyarrhythmia, pause, and bradycardia. However, interpreting ECG or ICM subcutaneous ECG (sECG) can be time-consuming. AI has shown promise in accurately classifying ECG and sECG data rapidly. Quartieri et al. proposed that AI algorithm could expand ICM arrhythmia recognition from four to a broader range of cardiac patterns [48]. The study showed that in 19 patients, ICMs recorded 2261 sECGs over an average follow-up period of 23 months. Among these 2261 sECG episodes, AI identified 7882 events and classified them into 25 different cardiac rhythm patterns with an overall accuracy of 88%. The AI also demonstrated strong positive predictive value (PPV) and sensitivity. It was particularly effective in identifying pauses, bradycardias, inverted T waves, and premature atrial contractions. Accordingly, the study found that AI can process sECG raw data from ICMs without prior training, thereby enhancing the performance of these devices and saving time for cardiologists in reviewing cardiac rhythm pattern detection [48]. Table 1 summarizes the studies on AI-assisted diagnostic tools for cardiovascular diseases.

**Table 1:** List of studies conducted on AI-assisted diagnosis in cardiovascular diseases AUC: area under the curve; CAD: coronary artery disease' ANN: artificial neural network; SPECT: single-photon emission computed tomography; ROC: receiver operating characteristic; RCA: right coronary artery; LAD: left anterior descending artery; LCX: left circumflex artery; ICM: ischemic cardiomyopathy; DCM: dilated cardiomyopathy; cMRI: cardiac magnetic resonance imaging; BED: biomarker extraction and discovery; WEKA: Waikato Environment for Knowledge Analysis; BRL: Bayesian rule learning; sECG: subcutaneous ECG; TNF-α: tumor necrosis factor alpha; IL-2: interleukin-2; NT-proBNP: N-terminal pro b-type natriuretic peptide; AI: artificial intelligence; MESA: Multi-Ethnic Study of Atherosclerosis

Study	Study Design	Methodology	Outcomes
Ross Upton, 2022 [38]	Prospective multicenter randomized crossover reader study	Evaluation of how availability of an AI classification might impact clinical interpretation of stress echocardiograms	Acceptable accuracy in identifying patients with severe CAD, heightened sensitivity in disease detection by 10% resulting in an AUC of 0.93, specificity of 92.7%, and a sensitivity of 84.4% enhance accuracy, inter-reader agreement, and reader confidence
Ying Guo, 2023 [41]	Prospective randomized controlled trial	The study included 818 patients undergoing coronary angiography, randomly divided into training (80%) and testing (20%) groups, with an additional 115 patients in the validation group. The study optimized a superior CAD diagnosis model using 59 echocardiographic features in a gradient-boosting classifier.	Characteristic AUC value of 0.852 in the test group and 0.834 in the validation group. High sensitivity (0.952) and low specificity (0.691) effectiveness in detecting CAD potential for increased false-positive
Hiroto Yoneyam, 2019 [42]	Prospective cohort study	The study included 59 patients diagnosed with stable CAD who had recently undergone both coronary angiography and myocardial perfusion SPECT imaging. The ability to identify culprit coronary arteries was evaluated for both experienced nuclear cardiologists and the ANN. This assessment was conducted using ROC curves and AUC analysis, allowing for a comparison of diagnostic accuracy between human experts and the AI system.	Diagnostic Accuracy: Observer A's accuracy with hybrid images: RCA: 83.6%, LAD: 89.3%, LCX: 94.4%; Observer B's accuracy: RCA: 72.9%, LAD: 84.2%, LCX: 89.3%; ANN's accuracy: RCA: 79.1%, LAD: 89.8%, LCX: 89.3%. Comparative Performance: the ANN demonstrated comparable diagnostic accuracy to experienced nuclear medicine physicians. Improvement with hybrid images: Significant enhancement in AUC for RCA region: Observer A: 0.715 to 0.835 (p = 0.0031), Observer B: 0.771 to 0.843 (p = 0.042). Challenges: Identifying culprit coronary arteries from perfusion defects in the inferior wall without hybrid images was difficult due to individual variations in LCX and RCA perfusion areas.
Mei Zhou, 2023 [46]	Retrospective study	The study analyzed echocardiogram data from 399 patients (200 with DCM, 199 with ICM) who underwent angiography between 2016 and 2022 at a single hospital. An external validation group of 79 patients was included. Data were randomly split and analyzed using four machine-learning methods. Cross-validation was conducted within the primary cohort, and the external cohort tested model generalizability, enhancing the study's validity and potential clinical applicability.	XGBoost emerged as the best-performing method, achieving an AUC of 0.934, 72% sensitivity, 78% specificity, and 75% accuracy in the primary cohort. In external validation, it maintained robust performance with an AUC of 0.804, 64% sensitivity, 93% specificity, and 78% accuracy. The model demonstrated high discriminative ability, correctly identifying ICM with 72% sensitivity and DCM with 78% specificity.
Vanathi Gopalakrishnan, 2015 [47]	Retrospective study	The researchers developed and tested cMRI-BED, a novel informatics framework for biomarker extraction and discovery from complex pediatric cMRI data, applying it to 83 de-identified cases and controls to classify cardiomyopathy findings in children. The framework incorporates image processing, marker extraction, and predictive modeling tools, utilizing Bayesian rule learning for interpretable models and machine learning methods from the WEKA toolkit for performance assessment using accuracy and AUC measures	The BRL decision tree model achieved the best predictive performance with 80.72% accuracy and 79.6% AUC in 10-fold cross-validation. Notably, the model identified myocardial delayed enhancement (MDE) status as an important predictive variable, aligning with its known clinical significance in cardiomyopathy classification.
Fabio Quartieri, 2023 [48]	Retrospective study	This study aimed to evaluate the capability of an AI algorithm to expand ICM arrhythmia recognition beyond the standard four cardiac patterns. To achieve this, researchers conducted an exploratory retrospective analysis using sECG data.	AI can process sECG raw data coming from ICMs without previous training, extending the performance of these devices and saving cardiologists' time in reviewing cardiac rhythm pattern detection.
Caiwei Zhang, 2020 [49]	Retrospective study	The study analyzed 14 characteristics of heart disease patients in Cleveland and Switzerland using various types of neural networks and classifiers to predict whether or not a patient has heart disease.	The logistic regression classifier performed better than other methods in predicting cardiovascular events.
Bharath Ambale-Venkatesh, 2017 [18]	Retrospective study	Used random survival forests, a machine learning method, to predict six different cardiovascular outcomes and compared its performance against traditional cardiovascular risk scores over 12 years. It included 6,814 participants (from the MESA) aged 45 to 84, with diverse ethnic backgrounds across the US, and focused on how early-stage disease progresses to cardiovascular events in initially healthy people	Imaging, electrocardiography, and biomarkers were more predictive than traditional risk factors. Age was consistently the strongest predictor for overall mortality. Fasting glucose levels and carotid ultrasound measurements were key for predicting strokes. The coronary artery calcium score stood out for predicting coronary heart disease and other related cardiovascular issues. Measures of left ventricular function and cardiac troponin-T were crucial for predicting heart failure. Creatinine levels, age, and ankle-brachial index emerged as top predictors for atrial fibrillation. Biomarkers like TNF-α, IL-2 soluble receptors, and NT-proBNP were important across all outcomes The random survival forests method outperformed traditional risk scores, improving prediction accuracy by reducing the Brier score by 10%–25%.

Prediction of cardiovascular events 

CVD is a leading cause of death worldwide, with various risk factors such as an unhealthy lifestyle, obesity, diabetes, and stress. Detecting and treating CVD early is crucial [50]. Kim et al. used a novel approach with a ML algorithm, SVM, to predict CVD at an early stage. They segregated CVD patients based on their symptoms and medical observations. The method aimed to help medical practitioners provide timely treatment. Consequently, it developed using this approach and has shown effective results in examining various stages of CVD compared to other ML techniques [50]. Furthermore, Zhang et al. created models and analyzed 14 characteristics of heart disease patients in Switzerland and Cleveland using various types of neural networks and classifiers [49]. The model based on these patient features was developed to predict whether or not a patient has heart disease. The study showed that the logistic regression classifier performed better than other methods in predicting cardiovascular events [13]. In addition, Ambale-Venkatesh et al. utilized ML to assess cardiovascular risk, predict outcomes, and find biomarkers in population studies [18]. The study used random survival forests, a ML method, to predict six different cardiovascular outcomes and compared its performance against traditional cardiovascular risk scores. The study involved 6,814 participants aged 45-84 years, from the Multi-Ethnic Study of Atherosclerosis (MESA) with diverse ethnic backgrounds. Researchers used baseline measurements to predict cardiovascular events over 12 years. MESA focuses on how early-stage disease progresses to cardiovascular events in initially healthy people. Surprisingly, imaging, electrocardiography, and biomarkers were more predictive than traditional risk factors. Age was consistently the strongest predictor for overall mortality. Consequently, the study showed using ML alongside detailed patient profiling enhances the accuracy of predicting cardiovascular events in initially healthy individuals. Table 1 summarizes the studies on diagnostic advances in CADs.

Challenges and limitations

The efficacy of AI models in cardiovascular imaging is heavily dependent on the quality and standardization of training data. Inconsistencies in image acquisition protocols, variability in equipment calibration, and differences in patient populations across healthcare institutions pose significant challenges [51]. These variations can lead to model overfitting or poor generalizability when applied to diverse clinical settings. Moreover, the lack of standardized labeling practices and the presence of noise or artifacts in medical images can compromise the accuracy of AI algorithms [52]. Efforts to establish multi-institutional databases and standardized imaging protocols are crucial but face logistical and regulatory hurdles [53].

Many advanced AI models, particularly deep learning architectures, operate as "black boxes," making it difficult for clinicians to understand the reasoning behind their outputs [54]. This lack of transparency can lead to skepticism and reluctance in clinical adoption [55]. Explainable AI (XAI) techniques, such as attention maps and feature importance analysis, are being developed to address this issue [56]. However, achieving a balance between model complexity and interpretability remains a significant challenge [57]. The ability to provide clear, justifiable explanations for AI-driven decisions is crucial for building trust among healthcare professionals and ensuring patient safety.

The integration of AI in cardiovascular imaging raises important regulatory and ethical concerns [58]. Regulatory bodies face the challenge of developing frameworks that ensure the safety and efficacy of AI algorithms without stifling innovation. Issues such as algorithm bias, data privacy, and informed consent need careful consideration. The potential for AI to perpetuate or exacerbate existing healthcare disparities is a growing concern [59]. Additionally, the question of liability in cases of AI-assisted misdiagnosis remains largely unresolved [60]. Striking a balance between innovation and patient protection requires ongoing dialogue between technologists, clinicians, ethicists, and policymakers.

Seamlessly incorporating AI tools into existing clinical workflows presents both technical and cultural challenges [9]. Many healthcare institutions lack the necessary IT infrastructure to support the deployment and maintenance of AI systems [61]. Integration with existing electronic health records (EHR) systems and picture archiving and communication system (PACS) can be complex and resource-intensive [53]. Furthermore, there is often resistance from healthcare professionals who may view AI as a threat to their expertise or autonomy [62]. Adequate training and education are essential to foster a collaborative approach between AI systems and human experts. Demonstrating tangible improvements in efficiency and patient outcomes is crucial for overcoming these barriers and achieving widespread adoption of AI in cardiovascular imaging.

Future directions

Emerging AI technologies in cardiovascular imaging are poised to revolutionize diagnostic accuracy and efficiency. Advanced deep learning architectures, such as transformer models and graph neural networks, show promise in analyzing complex imaging data with improved performance [63]. These technologies may enable more precise detection of subtle cardiovascular abnormalities and enhance risk stratification.

Multimodal imaging integration represents a significant frontier in AI-driven cardiovascular care. By combining data from various imaging modalities with clinical information, AI algorithms can provide a more comprehensive assessment of cardiovascular health [64]. This approach may lead to more accurate diagnoses and personalized treatment strategies.

AI in personalized medicine for CVDs is rapidly evolving. ML models are being developed to predict individual patient responses to therapies, optimize drug dosages, and identify patients at high risk for adverse events [9]. These advancements could enable tailored treatment plans that maximize efficacy while minimizing side effects. The potential for AI in population health management for cardiovascular diseases is substantial. Large-scale analysis of imaging data, combined with electronic health records and genomic information, could identify population-level trends and risk factors [65]. This could inform public health strategies and enable early interventions to reduce the burden of CVDs at a societal level.

As these technologies advance, it is crucial to address ongoing challenges such as data privacy, algorithmic bias, and clinical integration. Continued collaboration between clinicians, data scientists, and policymakers will be essential to realize the full potential of AI in cardiovascular imaging while ensuring ethical and equitable implementation [66].

## Conclusions

The integration of AI and ML in cardiovascular imaging represents a significant advancement in diagnostic capabilities and patient care. These technologies have demonstrated remarkable potential across various imaging modalities, including echocardiography, CT, MRI, and nuclear imaging. AI-assisted diagnosis has shown promise in detecting CAD, assessing valve disorders, classifying cardiomyopathies, identifying arrhythmias, and predicting cardiovascular events. The benefits of AI in this field include improved diagnostic accuracy, increased efficiency, and the potential for more personalized treatment approaches.

However, the implementation of AI in cardiovascular imaging is not without challenges. Issues such as data quality and standardization, model interpretability, regulatory and ethical concerns, and integration into existing clinical workflows must be addressed. As the field continues to evolve, future directions point towards more advanced AI architectures, multimodal imaging integration, and applications in personalized medicine and population health management. Overcoming these challenges and realizing the full potential of AI in cardiovascular imaging will require ongoing collaboration between clinicians, data scientists, and policymakers to ensure ethical, equitable, and effective implementation in clinical practice.
